# Genomic insights of *Salmonella* isolated from dry fermented sausage production chains in Spain and France

**DOI:** 10.1038/s41598-024-62141-9

**Published:** 2024-05-22

**Authors:** Núria Ferrer-Bustins, Claire Yvon, Belén Martín, Vincent Leclerc, Jean-Charles Leblanc, Laura Corominas, Sara Sabaté, Eva Tolosa-Muñoz, Carme Chacón-Villanueva, Sara Bover-Cid, Sabrina Cadel-Six, Anna Jofré

**Affiliations:** 1 IRTA, Food Safety and Functionality Programme, Finca Camps I Armet s/n, 17121 Monells, Spain; 2 Salmonella and Listeria Unit (SEL), Laboratory for Food Safety, ANSES, Pierre and Marie Curie Street 14, 94700 Maisons-Alfort, France; 3 LASPCAT_Girona, Public Health Agency, Department of Health, Government of Catalonia, Sol Street 15, 17004 Gerona, Spain; 4 Public Health Agency of Barcelona (ASPB), Lesseps Square 1, 08023 Barcelona, Spain; 5 Sant Pau Institute of Biomedical Research (IIB SANT PAU), Sant Quintí 77-79, 08041 Barcelona, Spain; 6 Surveillance Service, Food Control and Alerts Management, General Subdirectorate of Food Safety and Health Protection, Department of Health, Government of Catalonia, Roc Boronat Street 81-95, 08005 Barcelona, Spain; 7 Public Health Office, Department of Health, Government of Catalonia, Roc Boronat Street 81-95, 08005 Barcelona, Spain

**Keywords:** cgMLST, SNPs, Accessory genome analyses, Dry fermented sausages (DFS), *Salmonella*, Whole genome sequencing (WGS), Pathogens, Microbial ecology, Bacterial genetics, Food microbiology

## Abstract

The presence of *Salmonella* in dry fermented sausages is source of recalls and outbreaks. The genomic diversity of 173 *Salmonella* isolates from the dry fermented sausage production chains (pig carcasses, pork, and sausages) from France and Spain were investigated through their core phylogenomic relationships and accessory genome profiles. Ten different serovars and thirteen sequence type profiles were identified. The most frequent serovar from sausages was the monophasic variant of *S*. Typhimurium (1,4,[5],12:i:-, 72%) while *S*. Derby was in pig carcasses (51%). Phylogenomic clusters found in *S*. 1,4,[5],12:i:-, *S*. Derby, *S*. Rissen and *S.* Typhimurium serovars identified closely related isolates, with less than 10 alleles and 20 SNPs of difference, displaying *Salmonella* persistence along the pork production chain. Most of the *S*. 1,4,[5],12:i:- contained the *Salmonella* genomic island-4 (SGI-4), Tn21 and IncFIB plasmid. More than half of *S*. Derby strains contained the SGI-1 and Tn7. *S*. 1,4,[5],12:i:- genomes carried the most multidrug resistance genes (91% of the strains), whereas extended-spectrum β-lactamase genes were found in Typhimurium and Derby serovars. *Salmonella* monitoring and characterization in the pork production chains, specially *S*. 1,4,[5],12:i:- serovar, is of special importance due to its multidrug resistance capacity and persistence in dry fermented sausages.

## Introduction

Europe is the world’s second largest producer of pork (22 million tonnes in 2022)^1^ after China and the biggest exporter of pork and products thereof^2^. *Salmonella* contamination is a persistent problem in the pork production chain of many European countries. In the European Union (EU) on 2021, food business operators (FBO) samplings of pig carcasses at slaughterhouse detected 1.4% of *Salmonella*-positive samples, with values above the EU mean for France (4.6%) and Spain (3.9%). Subsequently, data gathered by the EU Member States for “Meat and meat products from pigs” category showed proportions of *Salmonella* positive samples up to 0.82% for ready-to-eat (RTE) (with an average of 0.23%) and 1.5% for non-RTE (average of 2.1%)^3^. Recently, during 2020–2023, dry fermented sausages (DFS), classified as RTE meat products, were responsible of 22 alerts related with *Salmonella* contamination as reported in the Rapid Alert System for Food and Feed (RASFF), being France the most notifying country and Spain the most identified country within the notification alerts (9 out of 22)^4^. From those, five were specifically linked to “fuet” (Refs. 2020.2344, 2020.3378, 2021.2535, 2021.3787, 2023.2633), a low-acid DFS traditional from the northeast region of Spain, Catalonia, made from lean pork, fat, salt, and pepper^5^, and two out of these five were linked to salmonellosis outbreaks (i.e., 2020.3378 and 2021.3787).

The most frequently reported serovars in pigs, as a food-animal source, and associated with human salmonellosis due to consumption of pork and its thereof products in the EU in 2021^3^ were the monophasic variant of *S.* Typhimurium (1,4,[5],12:i:-, 28.2%), *S*. Derby (22.3%), *S*. Typhimurium (15.3%) and *S*. Rissen (6.6%). Although these serovars are closely related genetically at subspecies level (they belong all to *Salmonella enterica* subsp. *enterica*), they can differ significantly in their pathogenic potentials^6–8^. Furthermore, within the same serovar, clones with a higher virulence and resistance potential may exist. Indeed, pathogenicity is directly associated with resistance to antimicrobials, biocide or heavy metal and virulence profile, traits usually acquired through mobile genetic elements (MGE) (i.e., transposons, integrons and plasmids)^9^. Subsequently, the dissemination of these specific and emerging clones can be favoured by international goods trade and human travelling^10^.

Whole genome sequencing (WGS) is currently the most robust method used in surveillance, microbial trace-back investigation, source attribution and risk assessment of food-borne microorganisms^11–14^, including *Salmonella* strains and circulating clones. The two main WGS-typing techniques are single nucleotide polymorphism (SNP) or allelic based methods. In particular, core-genome single nucleotide polymorphisms (cgSNP) and core-genome multilocus sequence typing (cgMLST, with 3002 loci in the case of *Salmonella* spp.) are largely used for bacterial typing and phylogenomic analysis^12,15^. Elseways, the accessory genome analyses allow exploring the most variable part of the microbial pan-genome, comprising the vertically or horizontally transferred DNA incorporated in the bacterial chromosome or contained in plasmids^16^.

By analysing 173 *Salmonella* isolates from the pork production chain, and more particularly from the DFS production chain, from France and Spain collected in the 1997–2021 period, the present study aimed to characterize the circulating clones with a high potential for resistance and virulence. The dissemination of the prevalent clones was also considered within the pork sector (from farm to fork) and possible trade between France and Spain, two countries among the largest producers of DFS and pork in Europe.

## Results

### Description of *Salmonella* serovars isolated in the French pork production chain

**Figure 1:**
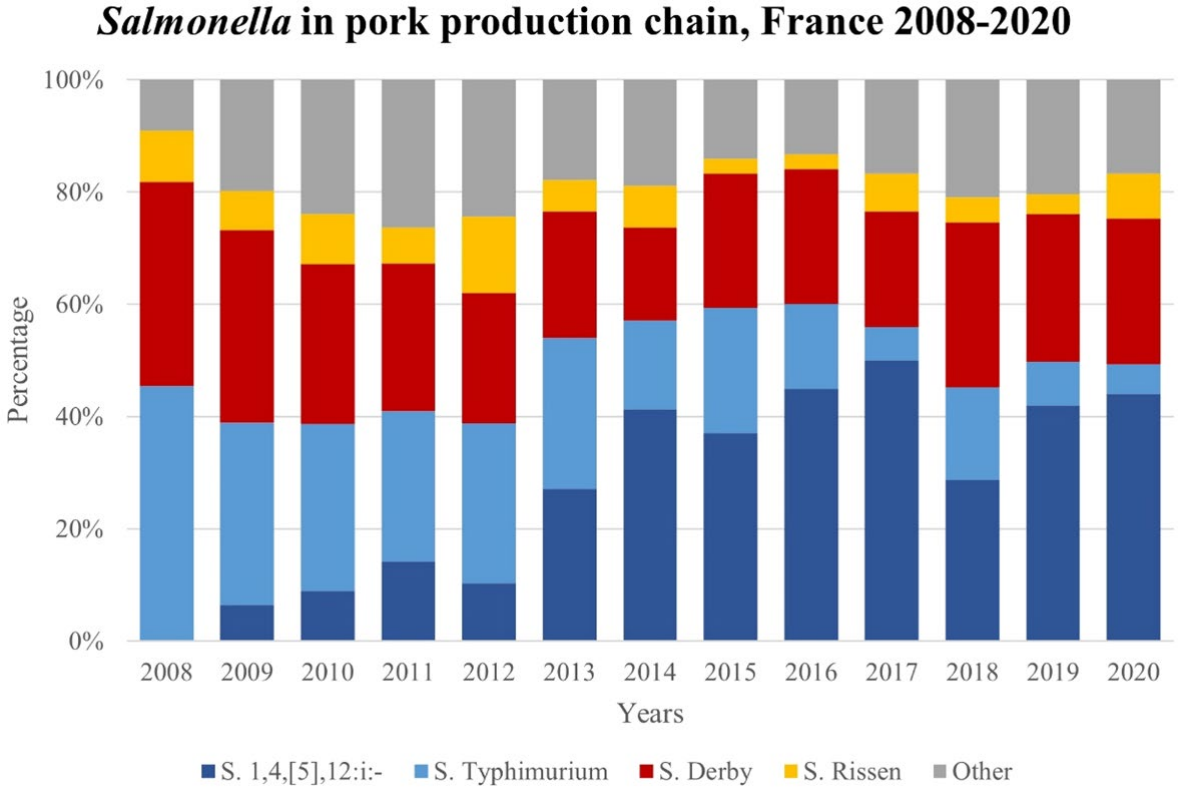
Serovar distribution of the main *Salmonella* serovars in the pork production chain in France from 2008 to 2020.

A total of 74 different serovars were identified among the 4717 *Salmonella* references of the French *Salmonella* Network collection from the pork production chain between 2002 and 2022 within the context of alerts, official control, surveys, surveillance, and control plans. Most of the references (97.9%) were isolated from 2008 to 2020 and, within this period, the main *Salmonella* serovars were *S*. 1,4,[5],12:i:-, *S*. Derby, *S*. Rissen and *S*. Typhimurium. Remarkably, *S*. 1,4,[5],12:i:- progressively increased from 2009 (6.4%) to become the most predominant serovar in the pork production chain in 2014 (41.3%) and then stabilized (Fig. 1). On the contrary, *S*. Typhimurium remarkably decreased its proportion from 2008 (45.5%) to 2020 (5.3%). From 2008 to 2020, there was a slight decrease in the proportion of *S*. Derby (from 36.4 to 26.0%) and *S*. Rissen (from 9.1 to 7.9%).

### Genome panel characteristics

Whole genome sequence data of 173 *Salmonella enterica* originating from pork and different stages of the DFS production chain in the northeast area of Spain and France were analysed. The sources of the strains were pig carcass (49), pork (38), fresh sausage (16) and pork DFS (70) (Table 1). A total of 125 isolates were collected during *Salmonella* surveillance, 31 in the context of outbreaks (27 specifically isolated from DFS), 14 and 3 were from IRTA and ANSES culture collections, respectively (Supplementary Table S1).

**Table 1:** Summary of the *Salmonella* serovars distribution (%) in the different matrixes studied (pig carcass, pork meat, fresh sausage, dry fermented sausages (DFS)) determined in silico using SeqSero+ and monophasic variant of *S*. Typhimurium confirmed by in silico PCR.

Serovar	Pig carcass	Pork Meat	Fresh sausage	DFS	Total isolates by serovar
*S*. 1,4,[5],12:i:-	29%	56%	100%	72%	102 (59%)
*S*. Derby	51%	42%	–	7%	45 (26%)
*S*. Rissen	14%	–	–	6%	11 (6%)
*S*. Typhimurium	4%	3%	–	7%	8 (5%)
*S*. Worthington	–	–	–	3%	2 (1%)
*S*. Goettingen	2%	–	–	-	1 (1%)
*S*. Infantis	–	–	–	1%	1 (1%)
*S*. Kedougou	–	–	–	1%	1 (1%)
*S*. London	–	–	–	1%	1 (1%)
*S*. Wien	–	–	–	1%	1 (1%)
Total isolates by matrix	49 (28%)	38 (22%)	16 (9%)	70 (40%)	173

Dash (–): Serovar not present in the panel.

Among the 173 *Salmonella* genomes analysed there were 10 different serovars: *S*. 1,4,[5],12:i:-, *S*. Derby, *S*. Rissen, *S*. Typhimurium, *S*. Worthington, *S*. Infantis, *S*. Kedougou, *S*. London, *S*. Wien and *S*. Goettingen (Table 1). *S*. 1,4,[5],12:i:- was the most prevalent serovar in DFS (72%), fresh sausages (100%) and pork (56%) and *S*. Derby was the most frequent in pig carcasses (51%).

*S*. Worthington, *S*. Infantis, *S*. Kedougou, *S*. London, *S*. Wien have only been detected in DFS (with only 1 or 2 isolates each).* S*. Goettingen has only been detected in pig carcasses from Spain.

### Multilocus sequence type (MLST) and cgMLST analysis

SeqSphere+ results revealed thirteen different MLST profiles in the 173 *Salmonella* genome panel including ST34 (58.4%) and ST5239 (0.6%) for *S*. 1,4,[5],12:i:-, ST40 (20.8%), ST39 (3.5%) and ST71 (1.7%) for *S*. Derby, ST469 (6.4%) for *S*. Rissen, ST19 (4.6%) for *S.* Typhimurium, ST9253 (1.2%) for *S*. Worthington, ST32 (0.6%) for *S*. Infantis, ST1543 (0.6%) for *S*. Kedougou, ST155 (0.6%) for *S*. London, ST9248 (0.6%) for *S*. Wien and ST20 (0.6%) for *S*. Goettingen (Supplementary Table S2, “ST_Summary” tab).

**Figure 2:**
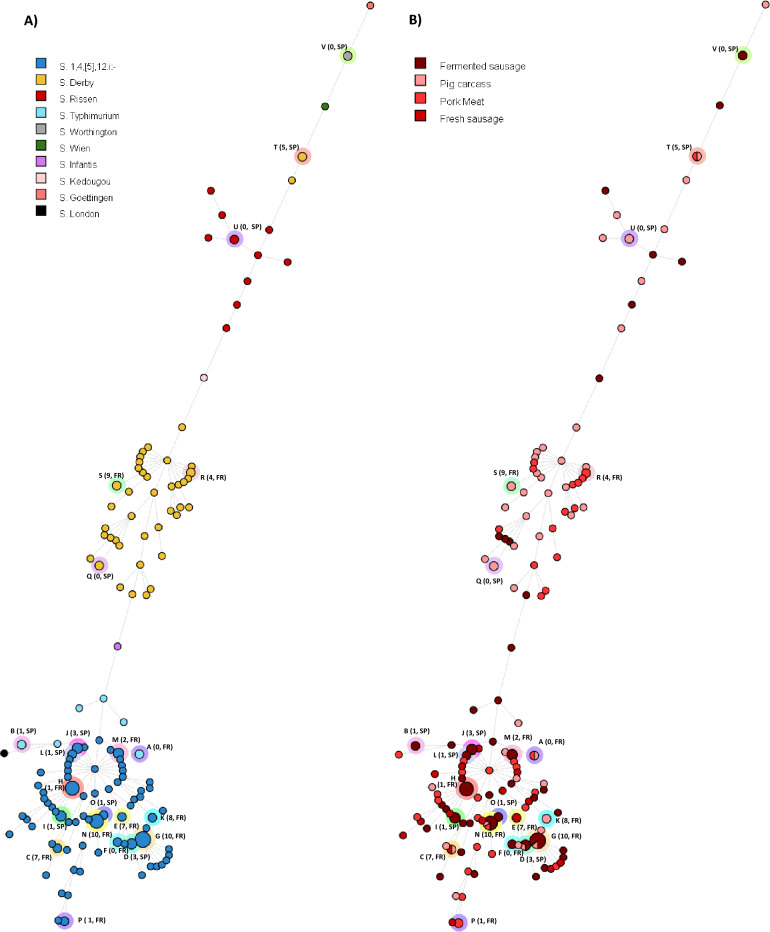
Minimum-spanning tree based on cgMLST analysis of the 173 *Salmonella* isolates. Each node represents a cgST. The node size is proportional to the number of isolates sharing the same genotype. The branch lengths correspond to allelic differences in log-scale. Clusters formed by nodes with a maximum of 10 allelic differences were labelled with coloured halos and, in parenthesis, the number of different alleles between the most distanced isolates in the cluster and the country of origin (SP: Spain; FR: France). Node colouring corresponds to the serovar in A and to the matrix type in B.

Considering the cgMLST results, a maximum likelihood phylogenomic tree (Supplementary Fig. S3) and a minimum spanning tree (Fig. 2) were built, and both clustered the isolates per each serovar except for *S*. Derby which had two different lineages due to its polyphyletic nature (ST39 and ST40 belonging to the lineage 1 and ST71 to the lineage 2)^17^.

Clustering association analysis revealed 22 clusters (named with the letter of the alphabet from A to V) using as cut-off a maximum of 10 alleles of difference between genomes and grouped 61 out of the 173 genomes (Supplementary Table S2, “cgMLST” tab). Within these 22 clusters, 2 belonged to *S*. Typhimurium (A-B), 14 to *S*. 1,4,[5],12:i:- (C-P), 4 to *S*. Derby (Q-T), 1 to *S*. Rissen (U) and 1 to *S*. Worthington (V) serovars (Fig. 2). Nine of the clusters included *Salmonella* isolated specifically from DFS (B, F, H, I, J, L, M, O, V), 4 from pig carcass (K, R, S, U), 2 from pork (P, Q),1 from fresh sausages (E), and the remaining 6 clusters included isolates from different matrixes. Two (D, G) out of 22 included DFS and pig carcass matrixes, 2 (A, S) pork and pig carcass, 1 (C) fresh sausage and pig carcass, and 1 (N, including six genomes) DFS, pork and pig carcass matrixes (Supplementary Table S2).

### SNP-based phylogenomic analysis

**Figure 3:**
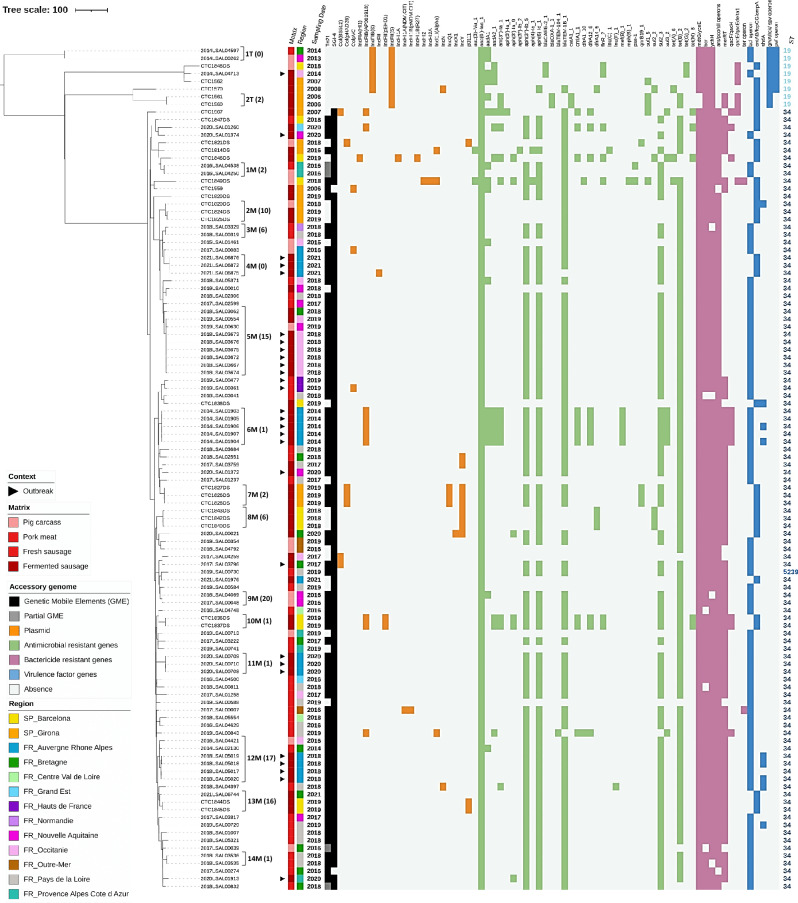
SNP core phylogenomic tree of 110 *S*. Typhimurium and *S*. 1,4,[5],12:i:- isolates including metadata, ST and accessory genome. Tree was constructed using LT2 reference genome. A cutoff of ≤ 20 SNPs highlighted 16 clusters indicated with numbers and letters (*e.g.*, 1T stands for Cluster 1 of *S*. Typhimurium and 1M stands for Cluster 1 of the monophasic variant, *S*. 1,4,[5],12:i:-) and, in parenthesis, the number of different cgSNPs between the most distanced isolates in the cluster. Outbreak related isolates are indicated with a black triangle. Matrix origin and geographic location are indicated with a coloured strip. Sampling year and sequence type (ST) are indicated as labels. Mobile genetic elements (black), plasmids (orange), antimicrobial resistance genes (green), biocide resistance genes (pink) and virulence factor genes (blue) are indicated as a heat map. The accessory genome genes that were found in all the isolates are not represented.

**Figure 4:**
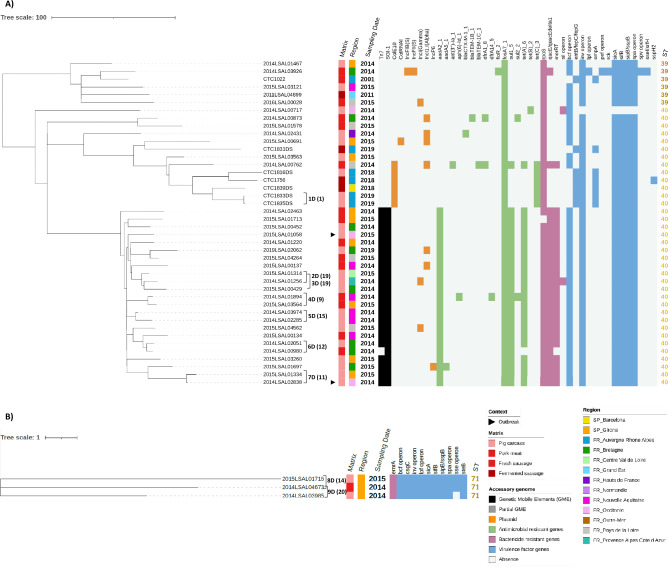
SNP core phylogenomic tree of 45 *S*. Derby isolates, including metadata, ST (ST39 and ST40 (**A**) and ST71 (**B**)) and accessory genome. Tree was constructed RM006 as reference genome A cutoff of ≤ 20 SNPs highlighted 9 clusters indicated with numbers and letters, *e.g.*, 1D stands for Cluster 1 of S. Derby, next to the strain label with the cgSNP value in parenthesis. Outbreak related isolates are indicated with a black triangle. Matrix origin and geographic location or region are indicated with a coloured strip. Sampling year and sequence type (ST) are indicated as labels. Mobile genetic elements (black), plasmids (orange), antimicrobial resistance genes (green), biocide resistance genes (pink) and virulence factor genes (blue) are indicated as a heat map. The accessory genome genes that were found in all the isolates of the *S*. Derby serovar are not represented in the figure.

**Figure 5:**
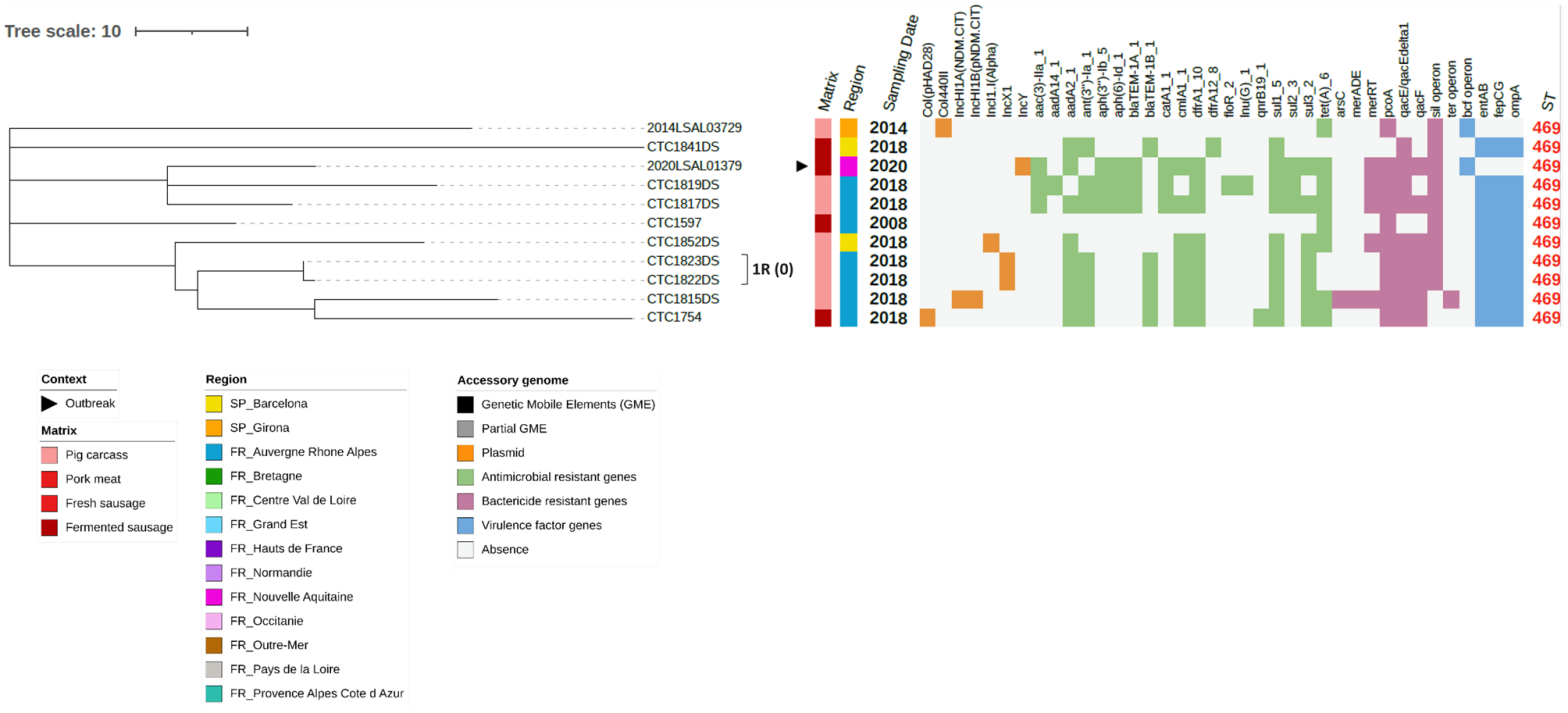
SNP core phylogenomic tree of the 11 S. Rissen including metadata, ST and accessory genome. Tree was constructed using GJ0703-2 as reference genome. A cutoff of ≤ 20 SNPs highlighted 1 cluster indicated with a number and letter, *e.g.*, 1R stands for Cluster 1 of *S*. Rissen, next to the strain label with the cgSNP value in parenthesis. Outbreak related isolates are indicated with a black triangle. Matrix origin and geographic location or region are indicated with a coloured strip. Sampling date, in years, and sequence type (ST) are indicated as labels. No mobile genetic elements were observed. Plasmids (orange), Antimicrobial resistance genes (green), biocide resistance genes (pink) and virulence factor genes (blue) are indicated as a heat map. The accessory genome genes that were found in all the isolates of the *S*. Rissen serovar are not represented in the figure.

The most abundant serovars (i.e., *S*. 1,4,[5],12:i:-,* S*. Typhimurium, *S.* Derby and *S.* Rissen accounted for 95.9% of the genomes analysed in this study) were further explored with the cgSNP phylogenomic analysis for epidemiological investigation and source tracking (see maximum likelihood phylogenomic trees in Figs. 3, 4, 5).

Using a cutoff of 20 cgSNPs, 26 clusters (labelled numerically and with letters according to the serovar, *e.g.*, 1T stands for Cluster **1** of *S*. **T**yphimurium) were identified according to the cgSNP phylogenetic results that grouped 68/173 genomes (Supplementary Table S2, “cgSNP” tabs). This clustering analysis was in accordance with the cgMLST results (Table 2) for *S.* Typhimurium (2 clusters), *S. *1,4,[5],12:i:- (14 clusters), and *S.* Rissen (1 cluster) serovars. In contrast, in *S*. Derby, 9 and 4 clusters were identified in the cgSNP and cgMLST analysis, respectively (Supplementary Table S2).

**Table 2:** Description of the phylogenomic clusters resulting from cgMLST (22 clusters, cutoff ≤ 10 alleles) and cgSNP (26 clusters, ≤ 20 SNPs) results encountered in *S.* Typhimurium *S. *1,4,[5],12:i:-, *S.* Derby, *S.* Rissen and *S*. Worthington serovars.

Serovar	Clusters		Total isolates	Metadata		
	cgMLST	cgSNP		Geolocation	Matrix origin	Sampling year
*S*. Typhimurium	A	1T	2	Nouvelle-Aquitaine, Bretagne	Pig carcass, pork	2013, 2014
	B	2T	2	Girona	Dry fermented sausage (DFS)	2006
*S. *1,4,[5],12:i:-	C	1M	2	Provence–Alpes-Côte-d’Azur	Pig carcass, fresh sausage	2016
	D	2M	3	Girona	Pig carcass, DFS	2018, 2019
	E	3M	2	Pays de la Loire, Normandie	Fresh sausage	2018
	F	4M	3	Auvergne-Rhône-Alpes	DFS	2021
	G	5M	7/9^a^	Occitanie, Nouvelle-Aquitaine/ Occitanie, Nouvelle Aquitaine, Bretagne^a^	DFS, pig carcass	2018, 2019
	H	6M	5	Auvergne-Rhône-Alpes	DFS	2014
	I	7M	3	Girona	DFS	2019
	J	8M	3	Barcelona	DFS	2018
	K	9M	2	Nouvelle-Aquitaine	Pig carcass	2016
	L	10M	2	Barcelona	DFS	2019
	M	11M	3	Auvergne-Rhône-Alpes	DFS	2020
	N	12M	6	Bretagne, Occitaine, Auvergne-Rhône-Alpes	Pig carcass, pork, DFS	2014, 2016, 2018
	O	13M	2/3^b^	Barcelona/Barcelona, Bretagne^b^	DFS	2019
	P	14M	2	Pays de la Loire	Pork	2018
*S*. Derby	R	1D	2	Nouvelle-Aquitaine, Auvergne-Rhône-Alpes	Pork	2014, 2015
	-	2D	2	Provence–Alpes-Côte-d’Azur, Centre Val de Loire	Pig carcass	2014, 2015
	-	3D	2	Centre Val de Loire, Bretagne	Pig carcass	2014, 2015
	Q	4D	2	Girona	Pig carcass	2019
	-	5D	2	Nouvelle-Aquitaine	Pig carcass	2014
	-	6D	2	Bretagne	Pig carcass, pork	2014
	S	7D	2	Occitanie, Auvergne-Rhône-Alpes	Pig carcass	2014, 2015
	T	8D	2	Auvergne-Rhône-Alpes	Pig carcass, pork	2014, 2015
	-	9D	2	Auvergne-Rhône-Alpes	Pig carcass, pork	2014
*S*. Rissen	U	1R	2	Girona	DFS	2018
*S.* Worthington	V	-	2	Girona	DFS	1997, 1999

Differences in the isolate metadata and accessory genome profile are indicated.

Underlined clusters share the same profile in the accessory genome (MGE, plasmids, AMR, virulence genes and biocide resistance genes).

^a^Seven isolates from Occitanie, Nouvelle Aquitaine clustered by cgMLST and 9 from Occitanie, Nouvelle Aquitaine, Bretagne by cgSNP.

^b^Two isolates from Barcelona clustered by cgMSLT and 3 from Barcelona and Bretagne by cgSNP.

Clusters formed by isolates sharing the same metadata and with a difference of ≤ 2 SNPs between isolates were formed by clonal isolates coming from the same sampling day, batch or belonging to the same outbreak. For *S*. 1,4,[5],12,:i:- serovar, six clusters (4M, 6M, 7M, 10M, 11M, 14M) shared the same metadata, for each *S*. Derby, *S*. Rissen and *S*. Typhimurium serovars, only one cluster grouped isolates sharing the same metadata and with ≤ 2 SNPs of difference between core genomes (1D, 1R, and 2T, respectively).

The largest cluster (5M) contained a total of nine closely related *S*. 1,4,[5],12,:i:- isolates, six of them isolated in 2018 from a DFS outbreak occurred in Occitanie. Only one cgSNP differed between the outbreak related isolates and the strain isolated in 2019 also from DFS in Occitanie, and there were six cgSNPs of difference with the strain isolated in 2019 from a pig carcass in Nouvelle-Aquitaine. In cluster 1T (identical cgSNP profiles), *S*. Typhimurium strains were isolated in 2013 and 2014 from a pig carcass and pork in Nouvelle-Aquitaine and Bretagne, respectively. Clusters 5M and 1T are examples of genotype persistence and survival along time in the pork sector in France.

Cluster 12M provided evidence of *S*. 1,4,[5],12:i:- genotype persistence in the French DFS production chain. The oldest strain of cluster 12M was sampled in 2014 in Centre-Val-de-Loire from pork, then another isolate was collected in 2016 in Occitanie from pig carcass, and four isolates in 2018 in Auvergne-Rhône-Alpes from outbreak related DFS. According to these results, this clone was circulating in the DFS production chain (slaughterhouse > cutting plant > retail) and different France regions for at least four years (2014–2018).

Other examples of clones circulating from pig carcasses to final sausage products are shown by the clusters 1M and 2M including region-specific isolates. Cluster 1M isolates were collected in November 2016 in the same region in France (Provence–Alpes-Côte-d’Azur), from both a pig carcass and a fresh sausage. Cluster 2M isolates were collected in the same region in Spain (Girona) with one strain isolated in 2018 from a pig carcass and two isolated in 2019 from DFS (same batch).

Clusters 4D, 7D (*S*. Derby ST40), and 8D (*S*. Derby ST71) isolates were collected in different years in pig carcasses and pork from the same or different geographic locations, and had few SNPs of difference, suggesting a common *S*. Derby ancestor circulating in the pork sector and within French regions.

Interestingly, the cluster 13M is an example of a multi-country occurrence with *S*. 1,4,[5],12:i:- isolates collected in Spain (Barcelona) in 2019 and in France (Bretagne) in 2021. The genomic distance within all the isolates is of 16 SNPs.

### Characterization of *Salmonella* isolates through accessory genome analysis

Resistome, virulome and MGE of all the genomes were examined to characterize the antimicrobial resistances, virulence potential, heavy metal, and biocide tolerances (Figs. 3, 4 and 5) (extended data in Supplementary Tables S4, S5 and S6).

#### Antimicrobial resistance genes

The antimicrobial resistance (AMR) gene analysis showed that all genomes possessed the cryptic aminoglycoside resistance gene *aac(6ʹ)-Iaa*. AMR prediction indicates that *S.* 1,4,[5],12:i:- serovar had a high variability in AMR genetic profile between isolates and more than the 91% of the isolates had multidrug resistance (MDR) genes. The *blaTEM-1B_1*, *aph(3″)-Ib_5* and *aph(6)-Id_1*, *sul2_3* and *tet(B)* genetic profile, described as conferring resistance to ampicillin (beta-lactamase), streptomycin (aminoglycoside), sulphonamide and tetracycline (ASSuT profile) typical of the epidemic clone was observed in 65.7% of the *S*. 1,4,[5],12:i:- genomes. A total of 10.8% isolates had the ACSSuTTm genetic profile which presents simultaneously the ASSuT profile and genes *cmlA1_1* and *dfrA12_8*, coding for chloramphenicol and trimethoprim resistances, respectively. Finally, 3.9% *S*. 1,4,[5],12:i:- isolates had a genetic profile *blaTEM-1B_1*, *aph(3″)-Ib_5* and *aph(6)-Id_1* and *sul2_3* coding for resistances to ampicillin, streptomycin and sulphonamide, respectively.

Fosfomycin resistance, *fosA7_1* gene, was only detected in *S*. Derby. The 55% of the *S*. Derby analysed genomes had the Tn7 and SGI-1 profiles, including *aadA2_1*, *sul1_5* and *tet(A)_6* genes, which code for resistance to aminoglycoside, sulphonamide, and tetracycline, respectively. These isolates closely clustered (ST40) and were recovered in both France and Spain from pig carcass and pork during 2014 and 2015. In contrast, *S*. Derby ST71 showed no AMR genes (except for *aac(6ʹ)-Iaa*) (Fig. 3).

Third generation extended-spectrum β-lactamase (ESBL) resistance genes were found in *S*. Typhimurium and *S*. Derby serovars. Specifically in *S*. Typhimurium, two isolates contained *blaCARB-2_1* and two other strains contained *blaOXA-1_1* ESBL genes, coding for resistance to carbenicillinase and carbapenemase, respectively. *blaCTX-M-1_1* ESBL resistance gene, coding for cefotaxime resistance^18^, was only found in one isolate of *S*. Derby ST40.

*S*. Rissen serovar showed a MDR genetic profile (81.8%) where the most frequent resistance genes were *sul1_5*, *aadA2_1*, *tet(A)_6* and *dfrA1_10*, coding for resistance to sulfamethoxazole, aminoglycoside, tetracycline, and trimethoprim, respectively.

#### Virulence genes

The virulence potential of the *Salmonella* isolates was evaluated through the presence of 141 virulence genes. Remarkably, a co-exclusion pattern was found in all the genomes: the presence of *bcf* locus (encoding for fimbrial related proteins) excluded the enterobactin related dehydrogenase and synthase genes (*entA* and *entB*, respectively), adhesin fimbriae genes (*fepC* and *fepG*) and outer membrane protein A (*ompA*) (Figs. 2, 3 and 4). The autotransporter *shdA* gene was found in *S.* Infantis (Supplementary Table S5) and unevenly found in *S.* 1,4,[5],12:i:- isolates from the same cluster (i.e., clusters D, H and N) (Fig. 2). The superoxide dismutase gene, *sodCl*, and the anti-inflammatory effector, *gogB,* were exclusively found in *S.* Typhimurium and *S.* 1,4,[5],12:i:- serovars. The type III secretion system genes, *sseI* and *srfH*, were found in *S.* Typhimurium (100%) and *S.* 1,4,[5],12:i:- (99%) and in one strain of *S.* Derby ST39 (Supplementary Table S5). *Salmonella* pathogenicity island (SPI) 3 (SPI-3) and 9 (SPI-9) were found in all the *Salmonella* isolates analysed. CG54 island was found in all *S*. Typhimurium, *S*. 1,4,[5],12:i:- and *S*. Infantis isolates, SPI-2 in all *S.* Typhimurium and *S.* 1,4,[5],12:i:- isolates and SPI-13 was exclusively found in *S.* 1,4,[5],12:i:- serovar. SPI-8 was found in all *S.* Rissen, *S.* Derby (ST71), *S.* Worthington, *S.* Kedougou, and *S.* Wien isolates (Supplementary Fig. S3, Supplementary Table S8).

#### Genes implied in biofilm formation, biocide and stresses tolerances

A total of 121 genes related to biofilm formation, stress adaptation and biocide and chemical/metal compounds resistance, among others, were evaluated (Supplementary Table S6). The profile of bactericide resistance genes highly depended on the serovar (Figs. 2, 3 and 4). All serovars carried 11 biocide resistance genes, five stress adaptation protein genes, and, apart from *S.* Goettingen, 10 biofilm formation genes. The *ars*/*pco*/*sil* operons, described to confer resistance to arsenic, cooper and silver, were simultaneously observed in 98% of *S.* 1,4,[5],12:i:- genomes, whereas *pco*/*sil* were simultaneously observed in 81.8% of *S.* Rissen isolates. Quaternary ammonium compounds resistance genes *qacE/qacEdelta1* (*qacEdelta1* is the truncated *qacE* and does not express itself) and *qacF/qacH* were present in *S.* Typhimurium (62.5%) and *S.* Derby (55.6%), and in *S.* Rissen (63.6%) and *S.* 1,4,[5],12:i:- (10.8%), respectively. *Ter* operon, linked to tellurite resistance, was detected in *S.* Rissen (9.1%) and *S.* 1,4,[5],12:i:- (2.0%). The three *S.* Derby ST71 isolates had the *emrA* gene, coding for an efflux pump associated to chromate resistance^19^*.* The truncated *mer* operon (*merRTPC*-Δ*merA*), involved in mercury resistance, was present in 1,4,[5],12:i:- (66.7%), Derby (51.1%) and Rissen (45.5%) serovars. The MGE Tn21 and SGI-4 were highly prevalent in *S*. 1,4,[5],12:i:- (80.4 and 98.0%, respectively) and Tn7 and SGI-1 were present in half of the *S*. Derby ST40 isolates (48.9% and 51.1%, respectively). In contrast, they were not detected among other serovar isolates.

#### Plasmids

The presence of plasmid replicons in *Salmonella* genomes was different among serovars (Supplementary Table S7). At least one plasmid was identified in *S*. 1,4,[5],12:i:- (33 genomes out of 102) (Fig. 2), *S*. Derby (16/45) (Fig. 3), *S*. Typhimurium (8/8) (Fig. 2), *S*. Rissen (7/11) (Fig. 4), *S*. Infantis (1/1), *S*. London (1/1) and, *S*. Wien (1/1) genomes, though no plasmids were identified in *S*. Goettingen, *S*. Kedougou and *S*. Worthington. IncFII(S) was detected in all *S*. Typhimurium and one *S*. Derby. Other Inc plasmid replicons were sparsely detected, being the most frequent IncFIB(S) in *S*. Typhimurium and, IncFIB(AP001918) and IncY in some *S*. 1,4,[5],12:i:-. However, the most abundant plasmid replicons in *S*. Derby were colE10 and IncI1.I(Alpha) (Figs. 2, 3 and 4). The presence of plasmid replicons was linked to phylogenomically related clusters H (IncFIB(AP001918), concurrently with the ACSSuTTm resistance genes profile) and I (col(pHAD28), IncQ1 and IncY) in the *S*. 1,4,[5],12:i:- serovar.

## Discussion

The relevance of *S*. 1,4,[5],12:i:- was raised in the recent decades and the serovar increase observed in France agrees with the information reported from Spain^20^ and worldwide^21^. *S.* 1,4,[5],12:i:- was first described as an atypical monophasic *S.* Typhimurium in 1987^22^ and it was spread in Spain during the 1990s^23^. From then on, within the context of pork industry globalization^24^, its dominance among the existing 2,600 *Salmonella* serovars has occurred in the pig herds specially^25–27^. *S.* 1,4,[5],12:i:- (ST34) is also the most abundant serovar from the evaluated panel of *Salmonella* genomes, corresponding to isolates from the pig production chain (i.e., pig carcasses, pork, pork sausages and dry fermented sausages) from both France (65%) and Spain (44%) during the 1997–2021 period.

Pig carcasses, before being cut, are cooled down to refrigeration temperatures (0‒4.4 °C), which has been reported to cause a *Salmonella* decrease in meat though not eliminating it completely^28^. Interestingly, the number of *S.* 1,4,[5],12:i:- isolates is higher in fresh sausages and DFS than in pig carcasses, its main source of contamination. These two facts could indicate that there is a selection towards the 1,4,[5],12:i:- serovar along pork and DFS production chain, a process which ends when food matrix is fermented (i.e., acidified) and dried^5^. DFS are a harsh environment for *Salmonella* and a progressive decrease of the pathogen has been described^29^. Under these circumstances, the high stress tolerance described for *S*. 1,4,[5],12:i:- among *Salmonella* serovars^30^ and the efficient colonization and survival abilities displayed above its parent *S*. Typhimurium strain^31^, could account for the higher prevalence of this serovar at the end of the pig production chain (i.e., fresh sausages and, particularly DFS). *S*. Derby and *S*. Rissen serovars seem less well adapted to the environment of DFS processing plants and to the production processes of DFS. Indeed, despite its prevalence during the last 20 years in pig herds has been stable^32^, as shown by French *Salmonella* Network data, our genomic panel showed a decrease of *S*. Derby and *S*. Rissen serovars along the DFS production chain, from pig carcass (51% and 14%, respectively) to the final product (7% and 6%, respectively).

The phylogenomic relationship between the 173 isolates shows *Salmonella* clusters of two or more isolates with equal or less than 10 allelic and 20 SNP differences in the core genome from *S*.1,4,[5],12:i:-, *S*. Derby, *S.* Rissen, *S*. Typhimurium and *S*. Worthington. Among the clusters, most of them indicated genotype persistence and survival along time in the pork sector and DFS production chain while others are related with region-specificity. Within our panel only three isolates clustered together in the cgSNP analyses although having different origin country, suggesting a witness of international trade exchange.

*Salmonella* Typhimurium and *S*. 1,4,[5],12:i:- isolates ancestry and phylogeny has been studied in several pig-related environments (i.e., pig farms and slaughterhouses)^20,33,34^ and only a few studies^35^ have focused in the production chain of pork products from official control sampling. The phylogenomic results have unveiled that 7 out of 9 *Salmonella* clusters identified within DFS matrix were due to the monophasic *S*. 1,4,[5],12:i:-, thus increasing its concern for official authorities and industry. *S*. Typhimurium short-term substitution rate has been reported to be of 1–2 SNPs per genome per year, thus providing information of strain clonality or common ancestor^36^. WGS-derived SNPs provided great cluster resolution in our panel that showed *S*. Typhimurium clonal isolates dissemination and transmission between regions (cluster 1 T, 0 cgSNPs) and *S*. 1,4,[5],12:i:- isolates with a common ancestor in the DFS production chain (cluster 12 M, 17 cgSNPs). Cross-country spread of *Salmonella* due to exportation of DFS was found in our phylogenetic results. Out of 173 samples analysed, three noticed cross-border contamination with a prevalence of 1.8% in our study. Other studies also reported *Salmonella* dissemination due to pig trade in Europe^20^.

In agreement with previous studies^37^, *S*. Derby STs mainly found in the French pork sector were ST40 and ST39 and the same was observed for Spanish genomes. Regardless of its polyphyletic nature, cgSNP analysis of *S*. Derby isolates was highly resolutive and closely related clusters were identified within ST40 and ST71 and genotypes with matrix or geographic persistence were shown. WGS approach has also been used for trace-back microbial investigations, which have indicated DFS as the main source of *Salmonella* outbreaks^38,39^. In our study, cluster 5M (15 cgSNPs) revealed seven 1,4,[5],12:i:- isolates from DFS related with an outbreak in 2018 and from pig carcass in 2019, which emphasizes the importance of following good manufacturing practices and validating the DFS production process^40^ together with adequate sampling plans and monitoring. Successful implementation of continuous monitoring of *Salmonella* has shown an effective control of the pathogen dissemination^41^. On the other hand, further studies should be carried out on the ability of some *S*. 1,4,[5],12:i:- clones to resist cleaning and disinfection practices applied in DFS manufacturing processes.

MGE determine the potential for genomic plasticity and pathogenicity of a bacteria^42,43^. Among the identified MGE, plasmid replicon types IncF and Col are the two most abundant replicon families in the dataset. IncFIB and IncFII virulence plasmids are among the best characterized and abundant plasmids within the genus^44^ and have been described to be part of the ancestral virulence plasmids together with *rck*, *spv* and *pef* virulence operons^45^. The inheritance of these plasmids is primarily vertical and serovar divergence theory may explain why only one *S*. Derby strain and all genomes of *S*. Typhimurium contain these plasmids^46^. Colicinogenic (Col) plasmids, which encode colicin bacteriocins, are typical from *Enterobacteriaceae* and are abundant in animal guts^47^. ColE10_1 is usually found in *Salmonella* and its relationship with quinolone resistance spread through *qnrS1* and *qnrB19* genes has been described^48^. In our panel, Col plasmids were found in genomes from *S*. 1,4,[5],12:i:- (9.80%), *S*. Derby (15.56%) and *S*. Rissen (18.18%), isolated from all studied matrixes. ColE10_1 plasmid was the most detected between the Col plasmids, specifically, it was found in 6 *S*. Derby genomes (13.33%), but *qnrB19_1* gene, that confers resistance to quinolone, was not detected concurrently.

Furthermore, transposons and *Salmonella* Genomic Islands are MGE usually integrated in the chromosome and carry specific antimicrobial resistance genes (ARG), virulence factors and biocide resistance genes. In 1980s, the acquisition of Tn21 and SGI-4 favoured the expansion of 1,4,[5],12:i:- European epidemic clone^49^. The majority of 1,4,[5],12:i:- genomes in the panel showed the Tn21 genetic element, which encodes mercury resistance (*merRT*) and antibiotic (ASSuT profile) genes, and SGI-4, encoding genes involved in arsenic (*ars* operon) and copper (*pco* operon) resistances^31,49,50^. Isolates of 1,4,[5],12:i:- mainly from DFS (90.9%) had the ACSSuTTm profile, which is usually related to the acquisition of the class 1 integron^51^. Stress conditions (*e.g.*, cleaning and disinfection procedures) promote the gain of MGE^52^ that can include genes conferring resistance to heavy metals, biocides and biofilm formation, providing the ability to overcome stress conditions and favouring *S.* 1,4,[5],12:i:- serovar survival and its selection^51,53^. In *S.* Derby ST40 there is a big cluster of isolates that carry resistance genes to quaternary ammonium and mercury compounds, co-occurring with *aadA2*, *sul1* and *tet(A)* AMR genes. This fact was already described by Sévellec et al.^17^ for the presence of the SGI-1, which also included *tetA* gene and extra mercury resistance genes (*merA* and *merC*) located in a Tn7 transposon.

*Salmonella* Typhimurium and its monophasic variant shared some genomic particularities (i.e., presence of SPI-2 and SPI-13) in comparison to the other studied serovars. SPI-2, which was found exclusively in *S.* Typhimurium and *S.* 1,4,[5],12:i:- isolates, is a 5-kb locus of horizontally acquired virulence genes that encodes a type III secretion system responsible for delivering effector proteins to the host cell after infection^54^. SPI-13, which was found in some genomes of *S. *1,4,[5],12:i:-, harbours genes that encode proteins putatively involved in bacterial metabolism, however, their functions remain largely uncharacterized^55^.

Virulence factors related to *Salmonella* adherence, *bcf* operon, and infection, *entAB*/*fepCG*/*ompA*, were excluding each other in the *Salmonella* genomes. The *bcf* gene, standing for bovine colonization factor, is an operon encoding for cryptic fimbriae and plays a role in the regulation of biofilm formation when *Salmonella* colonizes the intestines^56,57^, though has not been described to promote the biofilm formation in industrial surfaces. In contrast, *ent* operon encodes for the ferric iron binding siderophore enterobactin and *fep* operon encodes for the siderophore ABC transporter^58^. Both *ent* and *fep* operons, together with the ferric iron binding siderophore salmochelin constitute the primary ferric iron import system of *Salmonella* and are required for its persistent infection in macrophages^58^. Functions of outer membrane proteins (OMPs) are multiple and iron regulation function has also been attributed, specifically for the take up of ferri-siderophore complexes^59,60^. Nonetheless, *ompA*, encoding for the outer membrane protein A, plays an important role in the intracellular virulence of *Salmonella* due to the self-protection from the macrophages nitrosative stress^61^ and the activation of the immune system response^62^. The *shdA* gene was exclusively found in *S*. 1,4,[5],12:i:- and *S*. Infantis, unequally found in *S*. 1,4,[5],12:i:- isolates from the same sampling and in different proportions in the studied matrixes (7.1% in pig carcass, 5.0% in pork, 6.3% in fresh sausages and 11.5% in DFS). Gene *shdA* encodes for an OMP that is expressed while the pathogen inhabits the animal intestine and allows its specific binding through fibronectin^63^, an extracellular adhesion molecule involved in muscular tissue repair. The presence of *shdA* could be an advantage for *S*. 1,4,[5],12:i:- isolates attachment to pig carcasses and fresh pork, enhancing its selection along the production chain and together with the abovementioned stress tolerance result in the serovar persistence and survival.

Multidrug resistancet (MDR) *Salmonella* strains represent a serious challenge worldwide in the treatment and control of *Salmonella* infections, since these strains exhibit resistance to three or more antimicrobial classes^64^. MDR *Salmonella* isolates from pigs was of 39.1% in the EU in 2021^65^. Our results show that the most prevalent serovar in DFS, *S*. 1,4,[5],12:i:-, is also the serovar described to harbour more ARG in its genomes (i.e., 91% of *S*. 1,4,[5],12:i:- genomes had three or more ARG), thus proving the warning for its worldwide spread. Notwithstanding, extended-spectrum β-lactamase (ESBL) genes, *blaCARB-2_1* and *blaOXA-1_1*, were found in *S*. Typhimurium, and *blaCTX-M-1_1* in *S*. Derby ST40, in pig carcasses and DFS from both countries, France and Spain, since 2006. WGS approach allowed the detection of ESBL in a large genome dataset without in vitro susceptibility testing and the monitoring of MDR *Salmonella* profiles which is of interest for tracking resistome evolution and transfer in different ecosystems and to identify emerging resistance hazards more quickly^66^.

Several genetic markers of resistance to antibiotics and biocides, virulence factors and MGE have been found in the analysed *Salmonella* genomes, especially in *S.* 1,4,[5],12:i:-. Considering the high figures of the pig and pork derivatives industry and the fact that DFS are RTE products (i.e. eaten without the need for cooking), the transmission of *Salmonella* isolates and the corresponding resistance genes along the pork production chain is of concern. The ability of the enteric pathogen to survive along the DFS production process, overcoming disinfection cycles and DFS harsh conditions and the remarkable presence of strains with MDR genetic profile emphasize the need for *Salmonella* monitoring globally, paying special attention to *S*. 1,4,[5],12:i:- serovar. Further research on phenotype verification would confirm the survival advantage provided by the genetic markers encountered in the genomic *Salmonella* panel. In this context, WGS technology is a powerful tool to establish precise phylogenetic relationships between genomic clusters of persistent and transmissible strains in the pork sector, confirming the spread of the *S*. 1,4,[5]12:i:- European epidemic clone and characterizing the differences in the resistome and virulence profile between *Salmonella* serovars and food matrixes. Sharing genome sequences of isolates together with the corresponding metadata is essential to perform international pathogen surveillance, quickly identify outbreaks, and move forward towards the One Health approach.

## Materials and methods

### *Salmonella* isolates origin and selection

Fifty Spanish *Salmonella* isolates were analysed for this study, 36 isolates were provided by the official control food services of the Department of Health (Catalan Public Health Agency, Government of Catalonia) and 14 were from the IRTA culture collection. Isolates originated from different matrixes (pig carcass, n = 14, pork, n = 1, and DFS, n = 21) sampled in the frame of the “Biological Hazards Surveillance Program” (BHSP) and “*Salmonella* control program” (SCP), from 2016–2019 and 2018–2019, respectively. IRTA culture collection provided 12 *Salmonella* spp. genomes isolated from dry fermented sausages and 2 from pork from 1997–2018.

For French data, ANSES database of *Salmonella* Network was inquired for *Salmonella* spp. isolates collected from 2002 to 2022 from pig carcass, pork, and sausages (including DFS and “fresh sausages” made from pork and species). A total of 4717 references were obtained and evaluated to determine the proportion of the main serovars over time in the pork production chain in France. From those, 143 isolates had the genome available, and 123 genomes were selected for this study. For pig carcasses and pork origin genome isolates, sample duplicates were removed and the French regional pig carcass and pork production data^67^ was considered to balance the number of genome isolates for each French region (i.e., n = 35 genomes from pig carcasses and n = 35 from pork)^67^. All *Salmonella* genomes available from dry fermented sausages (n = 37) and fresh sausages (n = 16) were selected.

Specific information for Spanish and French *Salmonella* genomes, including matrix, is summarised in Supplementary Table S1.

### Genome sequencing and bioinformatics

Genomic DNA of the 50 IRTA *Salmonella* spp. isolates was extracted and isolated with the QIAamp DNA Mini QIAcube Kit (QIAGEN) with the automatic QIAcube sample preparation system (QIAGEN). DNA was quantified spectrophotometrically (µDrop plate, Thermo Fisher Scientific, Waltham, MA, USA) and fluorometrically (Quant-iT™ 1X dsDNA HS Assay Kit, Invitrogen, Merelbeke, Belgium) in a Varioskan™ multiplate reader (Thermo Fisher Scientific, USA). DNA samples were sent to Macrogen, Inc (South Korea) for library preparation and sequencing. Nextera DNA XT technology (Illumina) was used for library preparation and indexing according to the manufacturer recommendations. Paired-end sequencing (2 × 150 bases) was performed with an Illumina NovaSeq6000 sequencer.

The 123 French isolates were previously sequenced using Illumina chemistry producing paired-end reads as described by Radomski et al.^68^ and Sévellec et al.^17^.

Spanish and French raw reads were quality checked and filtered as described by De Sousa Violante et al.^69^ and with an in-house pipeline. In brief, Trimmomatic v0.40^70^ was used for the trimming step, FastQC v0.11.5 to check the read quality and ConFindr v0.8.1 to identify intra-and cross-species contamination^71^. An in silico PCR was performed to confirm the monophasic *S. *Typhimurium variant according to the primers described in the ISO/CD TS 6579-4^72^.

The metadata of the final panel of 173 *Salmonella* spp. genomes set for bioinformatic analysis are reported in the Supplementary Table S1.

### Phylogenomic analyses

All the genomes were uploaded and deposited in EnteroBase database (https://enterobase.warwick.ac.uk/). Whole genome sequences of 173 *Salmonella* spp. isolates were analysed trough in silico MLST, using the seven housekeeping loci (including *aroC, dnaN, hemD, hisD, purE, sucA* and *thrA*)^73^, and through cgMLST, using 3002^74^ loci based on SeqSphere+ v7.0.4 (Ridom R GmbH, Münster, Germany) scheme. A maximum likelihood phylogenetic tree was built considering the cgMLST SeqSphere+ results (Supplementary Fig. S3) and a minimum spanning tree was built with Bionumerics v7.6.3 (bioMérieux/Belgium) (Fig. 2).

For high-resolution genotyping, the cgSNP analysis was carried out by aligning the sequences of the most prevalent serovars (i.e., *S.* Typhimurium and its monophasic variant, *S.* Derby and *S.* Rissen), using snippy-core command, within Snippy v4.6.0 (https://github.com/tseemann/snippy). Reference strains used for cgSNP analysis were strains LT2 (NCBI NC_003197.1), RM006 (NCBI GCF_028892955.1) and GJ0703-2 (NCBI GCF_011057955.1), for the *S*. Typhimurium and its monophasic variant, *S.* Derby and *S.* Rissen serovars, respectively. The full-length whole-genome alignment was cleaned with the snippy-clean function and then used as an input to Gubbins v2.4.1 to filter and remove recombination artifacts^75^. The pairwise SNP differences were calculated using snp-dists v0.8.2 (https://github.com/tseemann/snp-dists). The alignment length for LT2 was 4,857,450 nucleotide and pairwise SNP differences ranged between 0 and 999. For RM006, the alignment length was 4,825,435 nucleotide sites and pairwise SNP differences ranged between 0 and 40,793. The alignment length for GJ0703-2 of 4,930,938 nucleotide sites and pairwise SNP differences ranged between 0 and 183.

The maximum likelihood phylogenomic trees were constructed from cgSNP results using RaxML v8.2.10^76^, with the evolutionary model GTRCAT and 100 bootstraps. Trees were visualized and annotated using interactive Tree Of Life (iTOL)^77^. A cutoff of 20 cgSNPs was set to define clusters of closely related isolates, based on the short-term substitution rate of 1–2 SNPs per genome per year for *Salmonella*^34,36,78^ and the range of strain isolation dates (1999–2021), as recommended by the European Food Safety Authority (EFSA) for *Salmonella* epidemiologically related strains^11^.

In silico detection of resistance and virulence genes, from ResFinder v4.4.2, Bacmet v2.0 and VFDB v4.0 databases was performed using an in-house pipeline whereas the detection of SPI and plasmid track down was performed through SPIFinder v2.0 and PlasmidFinder v2.0.1 databases, respectively, available online at the Center for Genomic Epidemiology (CGE) (Denmark). The minimum threshold of genetic identity was set at 90% for the in-house pipeline and 95% for the online databases and, the coverage at 80% in both cases.

### Supplementary Information

Supplementary Legends.

Supplementary Table S1.

Supplementary Table S2.

Supplementary Figure S3.

Supplementary Table S4.

Supplementary Table S5.

Supplementary Table S6.

Supplementary Table S7.

Supplementary Table S8.

## Data Availability

All genome sequences were deposited in EnteroBase database (https://enterobase.warwick.ac.uk/). Data is provided within the manuscript tables and figures and supplementary tables and figures.
