# Supplementary material for: Genomic insights of Salmonella isolated from dry fermented sausage production chains in Spain and France
Source: Sci Rep. 2024 May 22;14:11660. doi: 10.1038/s41598-024-62141-9 (PMC11111747; doi:10.1038/s41598-024-62141-9)
Supplement: Supplementary file 1 — Supplementary Legends. [file 41598_2024_62141_MOESM1_ESM.docx]

# Supplementary information

**Supplementary Table S1. Metadata information of the 173 Salmonella spp. genomes.** “STRAIN” states for the name of the isolate reference, “Collection” states for the name of the institution where the strain belongs (i.e., ANSES or IRTA), “Serovar” states for the *Salmonella* serovar identified (i.e., 1,4,[5],12:i:-, Derby, Goettingen, Infantis, Kegoudou, London, Rissen, Typhimurium, Wien or Worthington), “Sampling context” states for the context where the isolate was collected (i.e., surveillance, alert, official control or culture collection), “Matrix” states for the matrix where the isolate was originated (i.e., pig carcass, pork, fresh sausage or dry fermented sausage), “Place” states for the industry context where the isolate was collected (i.e., consumer’s residence, industrial processing, restauration collective, retail, slaughter or not identified), “Region” states for the province where the strain was isolated (i.e., Auvergne Rhone Alpes, Bretagne, Centre Val de Loire, Grand Est, Hauts de France, Normandie, Nouvelle Aquitaine, Occitanie, Outre-Mer (Guadeloupe), Outre-Mer (Réunion), Pays de la Loire or Provence Alpes Cote d Azur, for France, and Barcelona or Girona, for Spain), “Country” states for the country where the isolate was collected (i.e, France or Spain), “Collection_year” states for the year of isolate collection, and “Collection_month” states for the month of isolate collection.

**Supplementary Table S2. Sequence Type (ST), core-genome multilocus sequence type (cgMLST) and core-genome single nucleotide polymorphism (cgSNP) results for the 173 *Salmonella* spp. genomes.** In the “ST_Summary” tab there are the listed serovars, related with its ST, the number of genomes found and the proportion of each ST in the genome panel. In the “cgMLST” tab there are the isolates listed by strain name, related with its serovar, ST, metadata (i.e., Matrix, Region, Country, Collection_year, Collection_month) and the name of the cluster where the isolate was classified with less than 10 alleles of difference between genomes. In the “cgSNP_Typhi_mono”, “cgSNP_Derby” and “cgSNP_Rissen” there are the snp matrixes of *S.*Typhimurium and 1,4,[5],12:i:-, *S.* Derby and *S.* Rissen, respectively, with its reference strains (LT2 for *S.* Typhimurium and 1,4,[5],12:i:-, RM006 for *S.* Derby and GJ0703-2 for *S.* Rissen), and the name of the cgSNP cluster where isolates with less than 20 SNPs of difference were grouped.

**Supplementary Figure S3. Phylogenetic tree representation with all 173 Salmonella spp. genomes of the cgMLST.** The tree was constructed with a maximum likelihood of 100 bootstrap and rooted with S. Goettingen strain (CTC1839DS, ST20) as an uncommon serovar. Outer rings represent the presence (in dark grey) or absence (in light grey) of the different Salmonella Pathogenicity Islands (SPI), coloured numbers are the different sequence type (ST), and coloured branches indicate different serovars.

**Supplementary Table S4. Resfinder database results for the 173 *Salmonella* spp. genomes.** In the “resfinder” tab there is the resulting matrix of the antimicrobial resistant genes’ presence (1) or absence (0) for all genomes, relating them with the isolate name and serovar. In the “Typhimurium”, “Monophasic”, “Derby” and “Rissen” tabs, the antimicrobial resistant genes presence (1) or absence (0) are split between the corresponding serovars.

**Supplementary Table S5. Vfdb database results for the 173 *Salmonella* spp. genomes.** In the “vfdb” tab there is the resulting matrix of the virulent genes’ presence (1) or absence (0) for all genomes, relating them with the isolate name, country and serovar. In the “Typhi_mono”, “Derby” and “Rissen” tabs, the antimicrobial resistant genes presence (1) or absence (0) are split between the corresponding serovars. The rest of the tabs contain results information for each serovar.

**Supplementary Table S6. Bacmet database results for the 173 *Salmonella* spp. genomes.** In the “matrix_bacmet” tab there is the resulting matrix of the heavy metal and biocide resistant genes and biofilm formation and stress genes presence (1) or absence (0) for all genomes, relating them with the isolate name and serovar. In the “presence_bacmet_serotype_matrix”, it is indicated the number of genomes split per each serovar and matrix that contain the heavy metal and biocide resistant genes and biofilm formation and stress genes.

**Supplementary Table S7. PlasmidFinder database results for the 173 *Salmonella* spp. genomes.** In the “Plasmid_matrix” tab there is the resulting matrix of the plasmid presence (1) or absence (0) for all genomes, relating them with the isolate name, serovar, matrix and sampling context. In the “Typhi_mono”, “Derby” and “Rissen” tabs, it is indicated the number of genomes split per each serovar and matrix that contain the plasmid. In the “prevalence_plasmid_matrix_serov” tab there are the number of isolates that contain plasmids split by matrix and in the “prevalence_plasmid_serovar” tab there are the number of isolates that contain plasmids split by serovar.

**Supplementary Table S8. Salmonella Pathogenicity Island Finder (SPIFinder) database results for the 173 *Salmonella* spp. genomes.** In the “SPI” tab there is the resulting matrix of the SPI presence (1) or absence (0) for all genomes, relating them with the isolate name, serovar, matrix and sampling context. In the “prevalence_SPI_serovar” tab there are the number of isolates that contain SPI split by serovar and in the “prevalence_SPI_matrix_serovar” tab there are the number of isolates that contain SPI split by matrix and serovar.
