# Supplementary figures and images for: Genomic insights of Salmonella isolated from dry fermented sausage production chains in Spain and France
Source: Sci Rep. 2024 May 22;14:11660. doi: 10.1038/s41598-024-62141-9 (PMC11111747; doi:10.1038/s41598-024-62141-9)

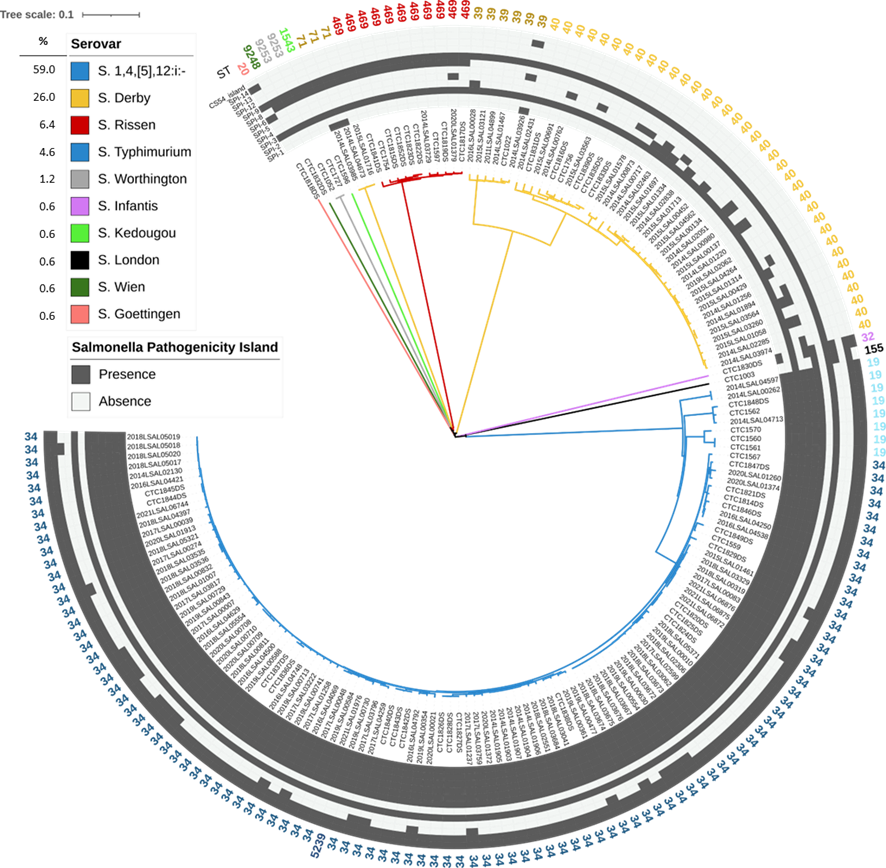

Supplement: Supplementary file 4 — Supplementary Figure S3. [file 41598_2024_62141_MOESM4_ESM.png]
